# Multi-Gene Phylogenetic Analyses Reveals *Heteroxylaria* Gen. Nov. and New Contributions to Xylariaceae (Ascomycota) from China

**DOI:** 10.3390/jof10090645

**Published:** 2024-09-11

**Authors:** An-Hong Zhu, Zi-Kun Song, Jun-Fang Wang, Hao-Wen Guan, Zhi Qu, Hai-Xia Ma

**Affiliations:** 1 Hainan Key Laboratory of Tropical Microbe Resources, Institute of Tropical Bioscience and Biotechnology, Chinese Academy of Tropical Agricultural Sciences, Haikou 571101, China; 18289679317@163.com (A.-H.Z.); michellesong2021@yeah.net (Z.-K.S.); 15379730137@163.com (J.-F.W.); 17725357096@163.com (H.-W.G.); quzhi@itbb.org.cn (Z.Q.); 2 School of Ecology and Nature Conservation, Beijing Forestry University, Beijing 100083, China; 3 Coconut Research Institute, Chinese Academy of Tropical Agricultural Sciences, Haikou 571101, China; 4 College of Plant Protection, Jilin Agricultural University, Changchun 130118, China; 5 School of Life Science, Liaoning University, Shenyang 110036, China; 6 Haikou Key Laboratory for Protection and Utilization of Edible and Medicinal Fungi, Hainan Institute for Tropical Agricultural Resources, Haikou 571101, China; 7 Chongzuo Key Laboratory for Protection and Utilization of Edible and Medicinal Fungi, Sanya Research Institute, Chinese Academy of Tropical Agricultural Sciences, Chongzuo 532100, China

**Keywords:** multigene phylogeny, ecological characters, new species, fructicolous-fungi, seminicolous-fungi

## Abstract

An in-depth study of the phylogenetic relationships of *Xylaria* species associated with nutshells of fruits and seeds within the genus *Xylaria* and related genera of Xylaceaecea was conducted in China. The multi-gene phylogenetic analyses were carried out based on ITS, RPB2, and TUB sequences of 100 species of 16 known genera in Xylariaceae around the world. Based on molecular phylogenetic analyses, morphological observations, and ecological habitats, a new genus, *Heteroxylaria*, is established to accommodate four new species, viz. *H. cordiicola*, *H. juglandicola*, *H. meliicola*, and *H. terminaliicola*, and four new combinations, viz. *H. oxyacanthae*, *H. palmicola*, *H. reevesiae*, and *H. rohrensis*. The genus is characterized by cylindrical stromata with conspicuous to inconspicuous perithecial mounds, surface black, having brown to dark brown ascospores with a germ slit, and it grows on nutshell of fruits. The combined ITS+RPB2+TUB sequence dataset of representative taxa in the Xylariaceae demonstrate that *Heteroxylaria* is grouped with *Hypocreodendron* but forms a monophyletic lineage. All novelties described herein are morphologically illustrated and compared to similar species and phylogeny is investigated to establish new genera and species.

## 1. Introduction

The family Xylariaceae Tul. and Tul. was introduced and typified by *Xylaria* Hill ex Schrank, and widely distributed in tropical, subtropical, and temperate regions [1,2,3,4,5,6]. Most taxa in the family are widely known as saprobes [4,7,8], while some are plant pathogens causing diseases of economic crops [9,10,11], and some are endophytes which have the potential to become pathogens when their hosts are stressed [3,12]. Many xylariaceous taxa are important producers of novel bioactive compounds and secondary metabolites [13,14,15,16]. Therefore, the xylariaceous taxa are of great interest due to their ecological and economic significance.

The number of genera accepted in Xylariaceae changed over the years. Based on morphologic characters in the 21th century, Eriksson and Hawksworth (1993) [17] accepted 35 genera but did not recognize *Nemania* and *Obolarina*, Læssøe (1994) [18] recognized 37 genera but included *Daldinia* and *Versiomyces* to *Hypoxylon*, Whalley (1996) [3] included 40 genera and considered *Nemania* and *Daldinia* as separate genera, respectively, while Rogers et al. revised 41 genera of the Xylariaceae (https://mycology.sinica.edu.tw/Xylariaceae/, accessed on 11 November 1997) in 1997. Kirk et al. (2008) [19] listed 85 genera and 1343 species in the Dictionary of the Fungi. Recently, based on multigene phylogeny, morphology, and chemotaxonomy analyses, Wendt et al. (2018) [20] separated Hypoxylaceae from Xylariaceae within Xylariales, and Daranagama et al. (2018) [21] accept 37 genera of Xylariaceae by observation of type specimens. Hyde et al. (2020) [22] listed 32 genera in Xylariaceae and were followed by Wijayawardene et al. (2020) [23]. Konta et al. (2020) [24] introduced a new genus, *Neoxylaria*, on palms (Arecaceae) from Thailand.

The genus *Xylaria* Hill ex Schrank, the type species *X. hypoxylon* (L.) Grev., is one of the most complex and difficult genera in the Xylariaceae [25,26]. Multi-gene phylogenetic analyses reveal that classification of *Xylaria* in Xylariaceae appeared as paraphyletic [20,21,24,27,28,29,30]. Hsieh et al. (2010) [27] found that *Xylaria* taxa were distributed in three main clades (TE, HY, and PO) within Xylariaceae based on stromatal morphology and multilocus phylogenetic analyses. Of the three clades, except clade TE, *Xylaria* taxa formed subclades with other genera of Xylariaceae, e.g., with *Kretzschmaria* taxa in clade HY and with *Astrocystis*, *Discoxylaria*, *Stilbohypoxylon*, and *Amphirosellinia* in clade PO, and were intermingled along the cladogram [27]. Konta et al. (2020) [24] presented that delimitation of *Xylaria* taxa were restricted to their morphology and habitats. We found that *Xylaria* species associated with fallen fruits and seeds evolved independently within *Xylaria*, and the phylogenetic relationships of the fructicolous *Xylaria* taxa may be influenced by the texture of the fruits or seeds [31]. Hence, it is necessary to carry out additional collections and studies for the primary identification and classification of xylariaceous taxa.

The type genus *Xylaria* is the largest genus in the family, with more than 670 morphological species (Hyde et al., 2020) [22] and 879 epithets in the Index Fungorum (http://www.indexfungorum.org/names/names.asp, accessed on 30 January 2024). About 77 species were reported from China [31,32,33,34,35,36,37,38,39,40,41,42,43]. This study is a continuation of the series on fructicolous fungi in China [31]. In this study, we introduce a new genus *Heteroxylaria*, four new species, and four new combinations for Xylariaceae occurring on nutshells of fallen fruits with morphological and phylogenetic evidence. Detailed descriptions, illustrations, and notes for each taxon are provided.

## 2. Materials and Methods

### 2.1. Sample Collection and Morphological Study

The nutshells of fruits and seeds bearing xylariaceous stromata are collected in evergreen broad-leaved forests or gemperate deciduous broad-leaved forests of Nature Reserves and Forest Parks from Guizhou, Hainan, Jilin, and Yunnan provinces of China. The studied specimens are preserved at the Fungarium of Institute of Tropical Bioscience and Biotechnology, Chinese Academy of Tropical Agricultural Sciences (FCATAS, Haikou, China). The stromatal surface and perithecia were examined with a VHX-600E 3D microscope of the Keyence Corporation (Osaka, Japan). The microscopical characteristics were observed with an Olympus IX73 inverted fluorescence microscope (Tokyo, Japan) and the CellSens Dimensions Software 3.17 (Olympus, Tokyo, Japan). The microscopic procedure followed Ma et al. (2022) [31]. Freehand sections were prepared from dried stromata and mounted in water, Melzer’s iodine reagent, 5% (*w*/*v*) potassium hydroxide (KOH), 1% (*w*/*v*) sodium dodecyl sulfate (SDS), and India ink. When presenting the variation in ascus and ascospore sizes, 5% of measurements were excluded from each end of the range and given in parentheses. In the morphological description, L is for ascospore length (arithmetical average of all ascospores), W for ascospore width (arithmetical average of all ascospores), Q for variation in the L/W ratios between the measured ascospores, and (a/b) for a number of ascospores (a) measured from a number of specimens (b). Color codes and names follow Rayner (1970) [44].

### 2.2. DNA Extraction and Sequencing

Dried specimens were taken for total genomic DNA extraction using a cetyltrimethylammonium bromide (CTAB) rapid extraction kit (Aidlab Biotechnologies Co., Ltd., Beijing, China) according to the method of Song et al. (2022) [45]. Three DNA gene fragments, ITS, RPB2, and β-tubulin (TUB) regions were amplified with primer ITS5/ITS4 [46], fRPB2-5F/fRPB2-7cR [47], and T1/T22 [48], respectively. The PCR procedures for the three sequences followed Pan et al. (2022) [43]. All newly generated sequences were uploaded on GenBank (https://www.ncbi.nlm.nih.gov/genbank/, accessed on 13 July 2024) and listed in Table 1.

### 2.3. Molecular Phylogenetic Analyses

In addition to the newly generated sequences, additional sequence data were downloaded from GenBank following recent studies [20,24,27,31,51]. To infer the phylogenetic position of *Heteroxylaria* within Xylariaceae, a three-locus dataset of ITS, RPB2, and TUB including 100 species of 16 known genera (*Amphirosellinia*, *Astrocystis*, *Brunneiperidium*, *Collodiscula*, *Entoleuca*, *Euepixylon*, *Hypocreodendron*, *Kretzschmaria*, *Nemania*, *Neoxylaria*, *Podosordaria*, *Poronia*, *Rosellinia*, *Sarcoxylon*, *Stilbohypoxylon*, and *Xylaria*) within Xylariaceae and one *Clypeosphaeria* species from *Clypeosphaeriaceae* as ingroup taxa and two species from Hypoxylaceae (*Daldinia loculatoides* Wollw. and M. Stadler and *Hypoxylon fragiforme* (Pers.) J. Kickx f.) and Barrmaeliaceae (*Barrmaelia rappazii* Jaklitsch, Friebes and Voglmayr, and *Entosordaria perfidiosa* (De Not.) Höhn.), respectively, as outgroup taxa were used according to Hsieh et al. (2010) [27] and Konta et al. (2020) [24].

Each locus of the dataset was aligned separately using the MAFFT V.7 online server (https://mafft.cbrc.jp/alignment/server/, accessed on 25 May 2024) and optimized manually using BioEdit 7.0.5.3 [65] and ClustalX 1.83 [66]. Maximum likelihood (ML) and Bayesian inference (BI) algorithms are performed for phylogenetic analyses based on a concatenated data set of ITS, RPB2, and TUB (ITS-RPB2-TUB). The ML analysis was conducted by raxmlGUI 2.0 [67] with GTRGAMMA+G as a substitution model. The BI analysis was conducted by MrBayes 3.2.6 [68] with jModelTest 2 conducting model discrimination. Six simultaneous Markov chains were run for one million generations and trees were sampled every 1000th generations. Of all sampled trees, the first 25% were discarded as burn-in, and the remaining trees were used to calculate the posterior probabilities (PP) of each branch. Phylogenetic trees were viewed in FigTree version 1.4.2 [69].

## 3. Results

### 3.1. Phylogenetic Analysis

The combined 3-gene (ITS-RPB2-TUB) dataset included sequences of 117 fungal samples representing 105 taxa. The dataset had an aligned length of 2921 characters, of which 1248 characters were constant, 287 were parsimony unformative, and 1386 were parsimony informative.

**Figure 1:**
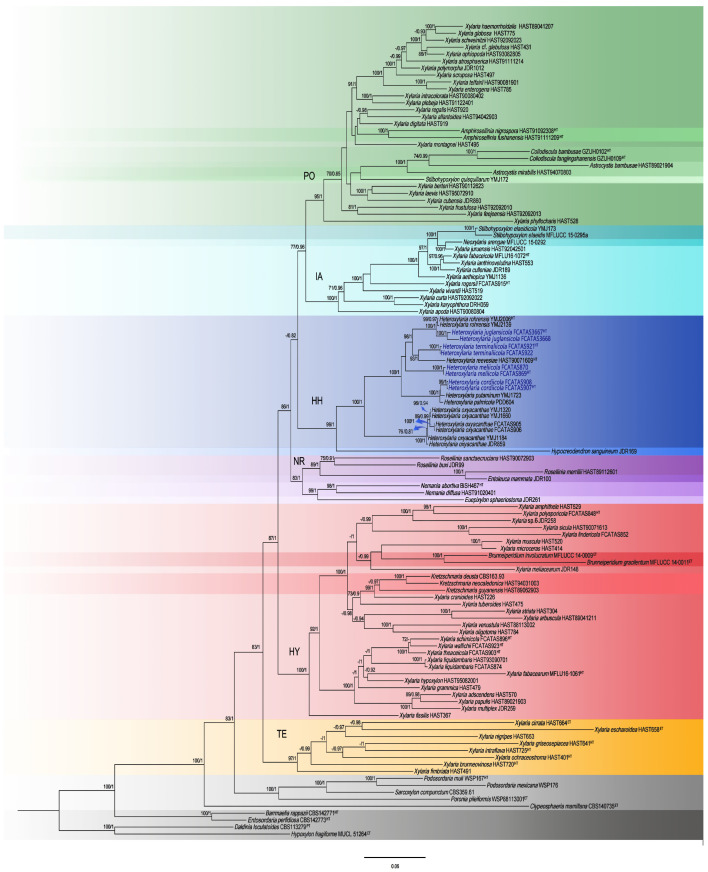
RAxML phylogenetic tree inferred based on the combined ITS-RPB2-TUB gene regions of selected species from Xylariaceae. ML bootstrap support (BS) ≥ 70% and Bayesian posterior probabilities (PP) ≥ 0.95 are given at the nodes in this order. New species in this study are indicated in blue.

The topology of multi-locus phylogenetic trees retrieved from BI and ML analyses are highly similar. The ML topology is presented along with BS values from the ML method and BPPs from the BI method, if simultaneously higher than or equal to 70% and 0.95 at the nodes, respectively (Figure 1). The phylogeny indicated that the new genus *Heteroxylaria* formed a distinct clade in the Xylariaceae but could not be included in any existing genera (Figure 1).

In the phylogenetic tree, *Poronia*, *Sarcoxylon*, and *Podosordaria* formed a strongly supported clade (100/1.00) separated early from other genera. *Amphirosellinia*, *Astrocystis*, *Brunneiperidium*, *Collodiscula*, *Entoleuca*, *Euepixylon*, *Hypocreodendron*, *Kretzschmaria*, *Nemania*, *Neoxylaria*, *Rosellinia*, *Stilbohypoxylon*, and the new genus *Heteroxylaria* clustered in six major clades together with species of *Xylaria*. The six clades, TE, HY, HH, NR, PO, and IA, were supported well and all of them received 100% posterior probability values and bootstrap values 97%, 100%, 99%, 83%, 95%, and 100%, respectively. The clade TE contained termite *Xylaria* species. Clade HY included the wood-inhabiting, foliicolous, and fructicolous species of *Xylaria*, and the taxa of *Brunneiperidium* and *Kretzschmaria*. Clade HH composed of species of the new genus *Heteroxylaria* and *Hypocreodendron* (*Discoxylaria*). Clade NR contained species of *Nemania*, *Rosellinia*, *Entoleuca*, and *Euepixylon*. Clade PO composed primarily of species of *Xylaria* growing on wood, and the species of *Amphirosellinia*, *Astrocystis*, *Collodiscula*, and *Stilbohyposylon quisquiliarum*. Clade IA composed primarily of species of *Xylaria* growing on fruits or legume pods, and a species of *Neoxylaria* and *Stilbohypoxylon*, respectively.

### 3.2. Taxonomy

***Heteroxylaria*** Hai X. Ma, A.H. Zhu and Y. Li, gen. nov.

**MycoBank:** MB854829

**Etymology:** Heteros = έτερος in Greek, other; similar in morphology but different from *Xylaria* in phylogeny.

**Type species:** *Heteroxylaria oxyacanthae* (Tul. and C. Tul.) Hai X. Ma, A.H. Zhu and Y. Li.

**Description:** *Sexual morph*: Stromata upright or prostrate, cylindrical, unbranched or branched; fertile parts cylindrical, with conspicuous to inconspicuous perithecial mounds; stipe definite to obscure, glabrous or tomentose, with longitudinally wrinkles, arising from a slightly enlarged base. Perithecia subglobose to obovoid, embedded in fertile parts. Ostioles papillate. Asci eight-spored, stipitate, with apical ring bluing in Melzer’s reagent. Ascospores brown, dark brown, unicellular, ellipsoid-inequilateral, with germ slit. *Asexual morph*: for a detailed description of *H. oxyacanthae*, see Stowell et al. (1983) [70].

**Notes:** Phylogenetically, specimens of *Heteroxylaria* formed a well-supported clade that is related to *Hypocreodendron*. Morphologically, *Hypocreodendron* differs by having upright stromata with an apical discoid to shallow cup bearing conidia and mature perithecia, fleshy to cartilaginous texture, brown ascospores ellipsoid with germ slit, and was found on ant nests [71]. In this study, *Heteroxylaria* is described as a new genus based on phylogenetic analyses, morphological characters, and ecological hatitats. Eight species are accepted in this genus including four new species, i.e., *H. cordiicola*, *H. juglandicola*, *H. meliicola*, and *H. terminaliicola*, and four new combinations, *H. oxyacanthae*, *H. palmicola*, *H. reevesiae*, and *H. rohrensis*. The genus is characterized by cylindrical stromata with conspicuous to inconspicuous perithecial mounds, surface black, brown to dark brown ascospores with germ slit, and it grows on nutshell of fruits.

**Figure 2:**
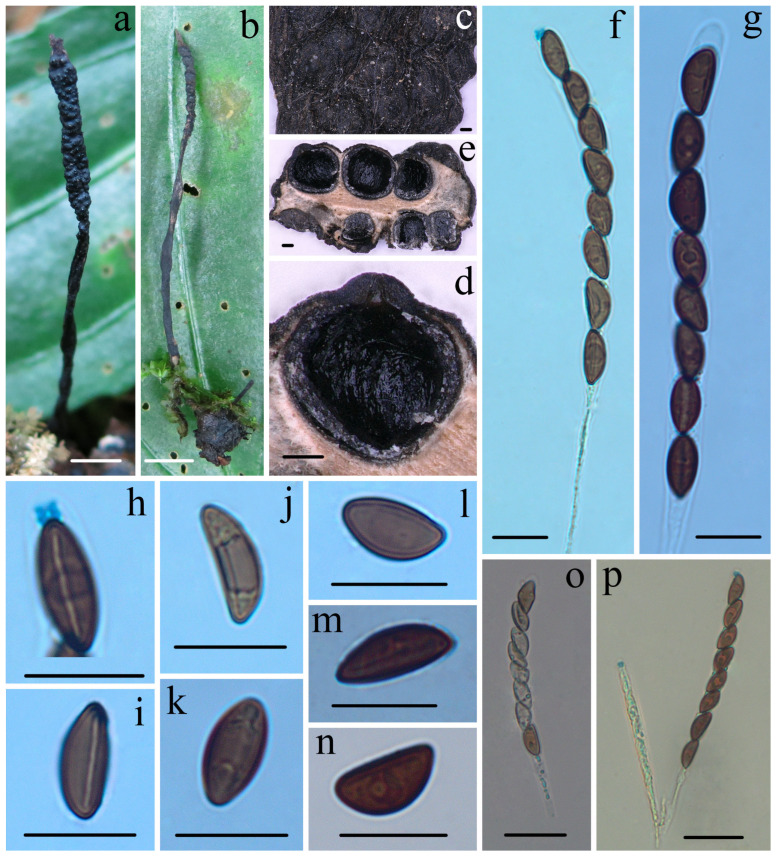
*Heteroxylaria cordiicola* (FCATAS 907, holotype). (**a**,**b**) Stroma on fallen fruit (**c**) Stromatal surface. (**d**,**e**) Section through stroma, showing perithecia. (**f**,**p**) Asci with ascal apical ring in Melzer’s reagent. (**g**) Asci in 1% SDS. (**h**) Ascal apical ring in Melzer’s reagent. (**i**) Ascospore with germ slit in Melzer’s reagent. (**j**,**l**) Ascospore in Melzer’s reagent. (**k**) Ascospore in 1% SDS. (**m**) Ascospore in India ink. (**n**) Ascospore in water. (**o**) Asci in water. Scale bars: (**a**,**b**) = 0.5 cm; (**c**–**e**) = 100 µm; (**f**–**n**) = 10 µm; and (**o**,**p**) = 20 µm.

***Heteroxylaria cordiicola*** Hai X. Ma, A.H. Zhu and Y. Li, sp. nov. Figure 2 and Figure 7d,e

**MycoBank:** MB854834

**Etymology:** *cordiicola* (Lat.): referring to the host genus *Cordia* which the fungus inhabits.

**Holotype:** CHINA: Guizhou Province, Libo County, Maolan Nature Reserve, on nutshells of fallen seeds of *Cordia dichotoma* G. Forst. (Boraginaceae), 16 July 2014, Ma Haixia, FCATAS907 (Col.135), GenBank numbers: ist: MZ648852, RPBS: MZ707116, TUB: MZ695791, LSU: MZ703211.

**Description:** *Sexual morph*: *Stromata* upright, simple, unbranched or occasionally branching, 3.0–7.5 cm total height, long stipitate; fertile parts 0.5–20 mm high × 0.8–2.0 mm broad, narrowly fusiform to cylindrical with acute sterile apices up to 2 mm long, at times furrowed, strongly nodulose with deep wrinkles isolating small groups of perithecia; stipes 2–50 mm high × 0.3–1.2 mm broad, glabrous to tomentose, somewhat flattened, with longitudinally wrinkles, arising from a slighly enlarged base. Surface black, with gray peeling outer layer and conspicuous perithecial mounds, continuous, glabrous; interior white to pale yellow, solid, and woody. Texture hard. *Perithecia* subglobose to obovoid, 280–450 × 300–500 µm. *Ostioles* conic-papillate. *Asci* eight-spored arranged in uniseriate manner, cylindrical, long-stipitate, (100–)115–135(–145) µm total length, the spore-bearing parts (65–)70–78(–84) µm long × (6.7–)7.0–8.0(–8.2) µm broad, the stipes 30–65 µm long, with apical ring bluing in Melzer’s reagent, urn-shaped to more or less rectangular, 1.5–2.0 µm high × 2.0–2.5 µm diam. *Ascospores* brown to dark brown, unicellular, ellipsoid-inequilateral with broadly rounded ends, sometimes with pinched on one end, smooth, (9–)10–11.7(–13) × (4.4–)5.0–6.0(–6.5) µm (M = 10.8 × 5.3 µm, Q = 2.0, n = 60/2), with a conspicuous straight germ slit full-length or nearly so, lacking a hyaline sheath or appendages visible in India ink or 1% SDS. As*exual morph*: not observed.

**Additional specimens examined:** CHINA: Guizhou Province, Maolan Nature Reserve, on nutshells of fallen seeds of *C. dichotoma* (Boraginaceae), 16 July 2014, Ma Haixia, FCATAS908 (Col.138), GenBank numbers: ITS: MZ648853, RPBS: MZ707117, TUB: MZ695792, LSU: MZ703212.

**Notes:** *Heteroxylaria cordiicola* resembles *Xylaria psidii* J.D. Rogers and Hemmes in stromatal morphology, but the latter species has cylindrical stromata with inconspicuous perithecia mounds and long acute sterile apex, smaller perithecia 200–300 μm, slightly smaller ascospores (7.5–)8–11(–12) × 4.5–5 μm, with a straight to slightly sigmoid germ slit [56,72], and grows on seeds of *Psidium guajava* L. (Myrtaceae). *Heteroxylaria cordiiacola* is somewhat similar to *H. oxyacanthae* in stromatal morphology, but the latter species has a paler peeling stromatal layer, long tomentose stipes, longer spore-bearing portion 65–100 μm, larger inverted hat-shaped apical ring 2.0–2.5 × 3.0 μm, and grows on mummified seeds of *C. monogyna* (Rosaceae) [70,73]. Moreover, *H. cordiiacola* formed its lineage and was not closely related to *H. oxyacanthae* in the phylogenetic tree (Figure 1). Although the phylogenetic analyses (Figure 1) show that *H. cordiiacola* has a close relationship with *H. palmicola* (*X. palmicola* Winter) and *Heteroxylaria* sp. (*X. putaminum* Maire and Durieu), *H. palmicola* is distinct morphologically for its longer stromata, larger ascospores (13.5–)14.5–16.5(–18.5) × (6–)6.5–7.5(–8.5) µm (M = 15.7 × 7.2 µm), and grows on *Euterpe* (Arecaceae) [56,74]. While *X. putaminum* (HAST145770) on buried stones of *Olea europea* var. *sylvestris* from Spain has a closer relationship with *H. palmicola* than *H. cordiiacola* in the phylogenetic tree, and sequence similarity of the species is higher with *H. palmicola* (ITS sequences 99.3%, RPB2 99.6%, and TUB 98.6%) than *H. cordiiacola* (ITS 98.37%, RPB2 99.32%, and TUB 97.44%). Morphologically, *Heteroxylaria* sp. (*X. putaminum*) differs from *H. cordiiacola* in having smaller stromata, larger ascospores 11.5–13.5 × 5–6(–6.5) µm [75].

**Figure 3:**
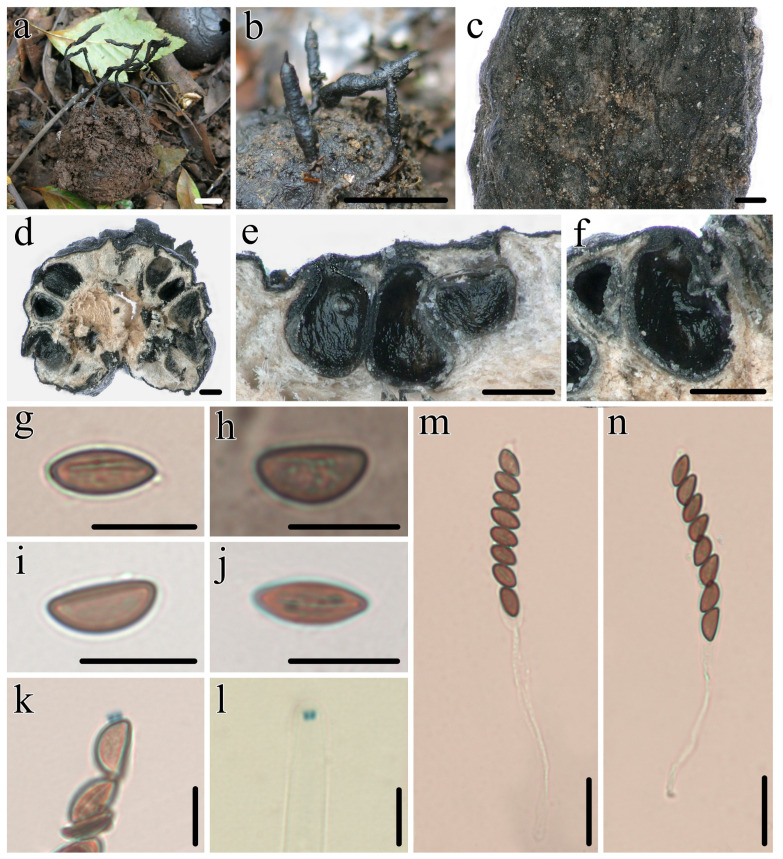
*Heteroxylaria juglandicola* (FCATAS 3667, holotype). (**a**,**b**) Stromata on fallen fruit. (**c**) Stromatal surface. (**d**–**f**) Section through stroma, showing perithecia. (**g**) Ascospore with germ slit in water. (**h**) Ascospore in India ink. (**i**) Ascospore in Melzer’s reagent. (**j**) Ascospore in 1% SDS. (**k**,**l**) Ascal apical ring in Melzer’s reagent. (**m**,**n**) Asci in water. Scale bars: (**a**,**b**) = 10 mm; (**c**–**f**) = 200 µm; (**g**–**l**) = 10 µm; and (**m**,**n**) = 20 µm.

***Heteroxylaria juglandicola*** Hai X. Ma, A.H. Zhu and Y. Li, sp. nov. Figure 3 and Figure 7a–c

**MycoBank:** MB854835

**Etymology:** *juglandicola* (Lat.): referring to the host genus *Juglans* which the fungus inhabits.

**Holotype:** CHINA: Yunnan Province, Yuxi City, Xinping County, Mopan mountain National Forest Park, on nutshells of fallen fruits of *Juglans regia* L. (Juglandaceae), 10 November 2019, Ma Haixia, FCATAS3667 (Col.M35), GenBank numbers: ITS: PQ009296, RPBS: PQ010279, TUB: PQ010278, LSU: PQ288591.

**Description:** *Sexual morph*: *Stromata* upright or prostrate, cylindrical, unbranched, with acute acute sterile apices, on long stipes originating from pannose bases, 2.0–6.5 cm total length; fertile parts cylindrical, 5.0–13 × 1.0–2.0 mm diam., slightly nodulose with wrinkles and finely longitudinally striate; stipes 0.5–6.0 cm length × 0.8–2 mm diam., well-defined, glabrous to tomentose, with a longitudinally furrowed, arising from a pannose, slightly enlarged base; surface black, with dark brown peeling outer layer and conspicuous perithecial mounds, glabrous; interior white to pale brown, solid, woody. Perithecia subglobose, 300–400 µm. Ostioles papillate. Asci eight-spored usually arranged in uniseriate manner, ascospores often overlapping, cylindrical, long-stipitate, (85–)90–115(–122) µm total length, the spore-bearing parts (45–)50–61(–66) µm long × (6.5–)7.0–8.3(–9.5) µm broad, the stipes 22–61 µm long, with apical ring bluing in Melzer’s reagent, wedge-shaped, 1.3–2.0 µm high × 2.0–2.8 µm diam. Ascospores brown, unicellular, ellipsoid-inequilateral with broadly rounded ends, occasionally with pinched on one end, smooth, (9.7–)10.5–11.7(–12.2) × (5.9–)6.0–7.0(–7.5) µm (M = 11.0 × 6.6 µm, Q = 1.8, n = 90/3), with a conspicuous straight germ slit less than a spore length, lacking a hyaline sheath or appendages visible in India ink or 1% SDS.

**Additional specimens examined:** CHINA: Yunnan Province, Yuxi City, Xinping County, Mopan mountain National Forest Park, on nutshells of fallen fruits of *J. regia* (Juglandaceae), 10 November 2019, Ma Haixia, FCATAS3668 (Col.N10), GenBank numbers: ITS: PQ009297, LSU: PQ288593; FCATAS3669 (Col.N31), GenBank numbers: LSU: PQ288592.

**Notes:** *Heteroxylaria juglandicola* is ascospores size similar to and phylogenetically closely related to *H. rohrensis* Friebes, A. Gallé, H.-M. Hsieh and Y.-M. Ju also growing on nutshells of *J. regia* (Figure 1) from Austria, but the latter species has stronger stromata, inverted hat-shaped and larger apical ring 2.5–2.8 µm high × 2.5–2.6 µm broad, the spore-bearing part longer 65–75 µm, brown to dark brown ascospores and a European distribution [57]. In addition, there is and eight-base-pair difference between the sequences of *H. juglandicola* and *H. rohrensis*, which accounts for 4.17% and 2.28% of the nucleotides in the ITS1 and ITS regions, respectively. Phylogenetic analyses show that *H. juglandicola* has a close relationship with *H. terminalliicola* and *H. reevesiae* (Figure 1). Morphologically, *H. terminalliicola* differs by having more or less cylindrical stromata with a mucronate or blunt sterile apex, an ill-defined stipe, tubular and larger an apical ring (2.2–3.8 µm high × 2.4–3.2 µm diam), a larger ascospores size range (10.0–)11–13.0(–13.8) × (5.2–)6.0–7.0(–7.8) µm (M = 12.0 × 6.7 µm), and it grows on nutshells of *Terminalia catappa* (Combretaceae). *Heteroxylaria reevesiae* is distinguished by cylindrical stromata with conspicuous perithecial mounds, brown and smaller ascospores (8.5–)9–10.5(–11) × (4–)4.5–5.5(–6) µm (M = 9.7± 0.6 × 5.0 ± 0.3 µm), and it grows on fallen fruits of *Reevesia formosana* (Sterculiaceae) [56].

**Figure 4:**
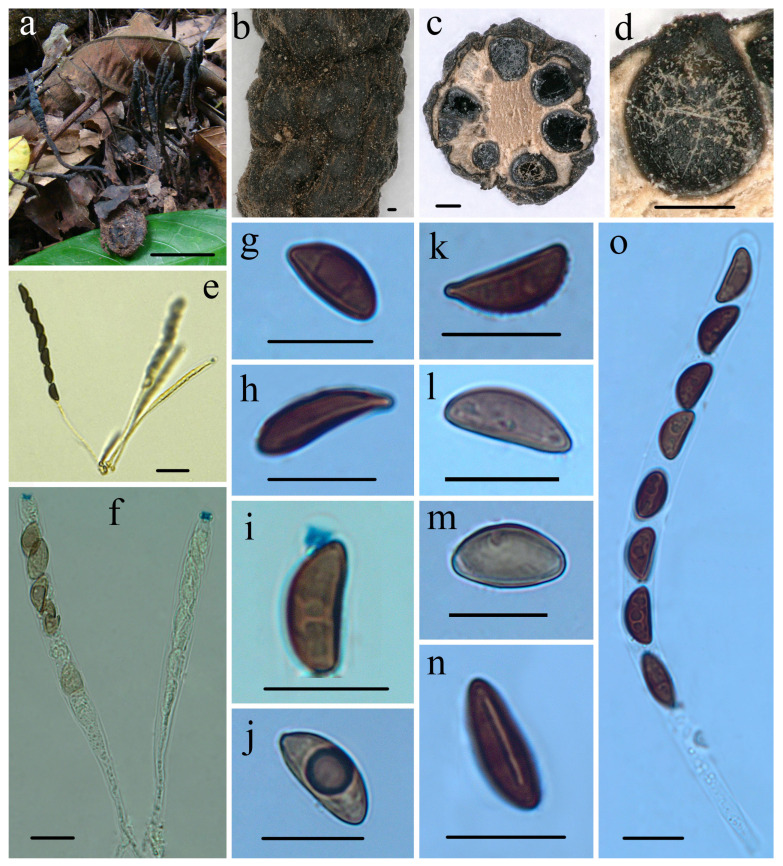
*Heteroxylaria meliicola* (FCATAS 869, holotype). (**a**) Stromata on fallen fruits. (**b**) Stromatal surface. (**c**,**d**) Section through stroma, showing perithecia. (**e**,**f**) Asci with ascal apical ring in Melzer’s reagent. (**g**) Ascospore in water. (**h**) Ascospore with beaked ends in water. (**i**) Ascal apical ring in Melzer’s reagent. (**j**) Ascospore in Melzer’s reagent. (**k**,**l**) Ascospore in India ink. (**m**) Ascospore in 1% SDS. (**n**) Ascospore with germ slit in 1% SDS. (**o**) Asci in 1% SDS. Scale bars: (**a**) = 2 cm; (**b**) = 100 µm; (**c**,**d**) = 200 µm; (**e**) = 20 µm; and (**f**–**o**) = 10 µm.

***Heteroxylaria meliicola*** Hai X. Ma, A.H. Zhu andY. Li, sp. nov. Figure 4 and Figure 7f,g

**MycoBank:** MB854836

**Etymology:** *meliicola* (Lat.): referring to the host genus *Melia* which the fungus inhabits.

**Holotype:** CHINA: Yunnan Province, Mengla County, Xishuangbanna Tropical Botanical Garden, on buried nuts of *Melia toosendan* Sieb. et Zucc. (Meliaceae), 20 October 2013, Ma Haixia, FCATAS869 (Col.9), GenBank numbers: ITS: MZ648845, RPBS: MZ707104, TUB: MZ695773.

**Description:** *Sexual morph*: *Stromata* upright or prostrate, cylindrical, terete to somewhat flattened, unbranched or occasionally branched, with acute sterile apices up to 5.5 mm, on long stipes originating from pannose bases, 4.5–8 cm total length; fertile parts cylindrical, 20–35 × 1.5–5 mm diam., with conspicuous perithecial mounds, overlain with wrinkles and finely longitudinally striate; stipes 20–45 mm length × 1–2.5 mm diam., well-defined, glabrous, with a longitudinally furrowed, arising from a pannose, slightly enlarged base; surface blackish with dark brown peeling outer layer, with immersed perithecia, interior white, often brown at center, solid, and woody. Texture hard. *Perithecia* subglobose, 300–500 µm. *Ostioles* faintly to papillate. *Asci* eight-spored usually arranged in uniseriate manner or occasionally in a partially biseriate manner, cylindrical, long-stipitate, (96–)110–125(–140) µm total length, the spore-bearing parts (60–)63–82(–88) µm long × (6.5–)7.0–8.0(–8.6) µm broad, the stipes 30–76 µm long, with apical ring bluing in Melzer’s reagent, urn-shaped to tubular, and 1.6–2.2 µm high × 1.5–2.0 µm broad. *Ascospores* brown to dark brown, unicellular, ellipsoid or pyriform, inequilateral, with narrowly to broadly rounded ends, smooth, aberrant ascospores with strongly pinched or beaked ends can be often encountered, (8.3–)9.5–12.0(–12.7) × (4.2–) 4.6–5.6(–6.2) µm (M = 10.4 × 5.1 µm, Q = 2.1, n = 90/3), with a conspicuous straight germ slit almost spore-length or less than spore-length, lacking a sheath or appendages visible in India ink or 1% SDS.

**Additional specimens examined:** CHINA: Yunnan Province, Mengla County, Xishuangbanna Tropical Botanical Garden, on buried nuts of *M. toosendan* (Meliaceae), 5 August 2010, Ma Haixia, FCATAS870 (Col.66), GenBank numbers: ITS: MZ648846.

**Notes:** *Heteroxylaria meliicola* is characterized by long cylindrical to irregular stromata with inconspicuous perithecial mounds and longitudinally striate, ellipsoid or pyriform ascospores with a straight germ slit, and grows on nuts of *M. toosendan* in Meliaceae. Læssøe and Lodge (1994) [76] described two *Xylaria* species on the Meliaceaea, *X. meliacearum* Læssøe and *X. guareae* Læssøe et Lodge. *Xylaria meliacearum* was found on leaf petioles and midveins of *Trichila* and *Guarea*, and *X. guareae* on the branches of *G. guidonia*. However, *H. meliicola* is distinctly different from the two species. *X. meliacearum* has strap-like stromata, stipitate unclear, separated from fertile part and larger ascospores (18.5–)19.1–30.0(–33.0) × (4.0–)4.6–6.6(–7.9) µm, whereas *X. guareae* has smaller stromata [2–6(–8) × 1.5–3(–6) mm], compressed obpyriform or pulvinate, and larger ascospores 39–50 × 13.6–17.0 µm [76].

Two species, *H. oxyacanthae* and *H. palmicola*, are somewhat similar to the Chinese collections in stromatal morphology, but *H. oxyacanthae* has a paler peeling stromatal layer, long tomentose stipes, larger inverted hat-shaped apical ring 2.0–2.5 × 3.0 μm, and grows on mummified seeds of *Crataegus monogyna* (Rosaceae) [56,70,73]. While *H. palmicola* differs in having larger ascospores (13.5–)14.5–16.5(–18.5) × (6–)6.5–7.5(–8.5) µm (M = 15.7 × 7.2 µm), and grows on fruits of *Euterpe* (Arecaceae) [56,74]. Moreover, *H. cordiiacola* formed its lineage and was not closely related to *X. meliacearum*, *H. oxyacanthae*, and *H. palmicola* in the phylogenetic tree (Figure 1).

Phylogenetically, *Heteroxylaria meliicola* grouped with *H. terminaliicola*, *H. reevesiae*, *H. juglandicola*, and *H. rohrensis* with no significant support (−% ML, − BPP). Morphologically, *H. meliicola* resembles *H. reevesiae* YM Ju, JD Rogers and HM Hsieh in stromatal morphology, but the latter species has cylindrical stromata with conspicuous perithecial mounds, slightly smaller ascospores (8.5–)9–10.5(–11) × (4–)4.5–5.5(–6) µm (M = 9.7 × 5.0 µm), and grows on fallen fruits of *Reevesia formosana* (Sterculiaceae) [56]. *Heteroxylaria terminaliicola* differs from *H. meliicola* in having stronger stromata with a mucronate or blunt sterile apex, larger apical ring 2.2–3.8 µm high × 2.4–3.2 µm diam, larger ascospores (10.0–)11–13.0(–13.8) × (5.2–)6.0–7.0(–7.8) µm (M = 12.0 × 6.7 µm), and grows on nutshells of *Terminalia catappa*. *Heteroxylaria juglandicola* differs by having smaller stromata, shorter in the spore-bearing part, and slightly larger ascospores, while *H. rohrensis* is distinguished by having smaller stromata, larger apical ring 2.5–2.8 µm high × 2.5–2.6 µm broad, inverted hat-shaped, and the two species grow on nutshells of *J. regia* (Juglandaceae) [57].

**Figure 5:**
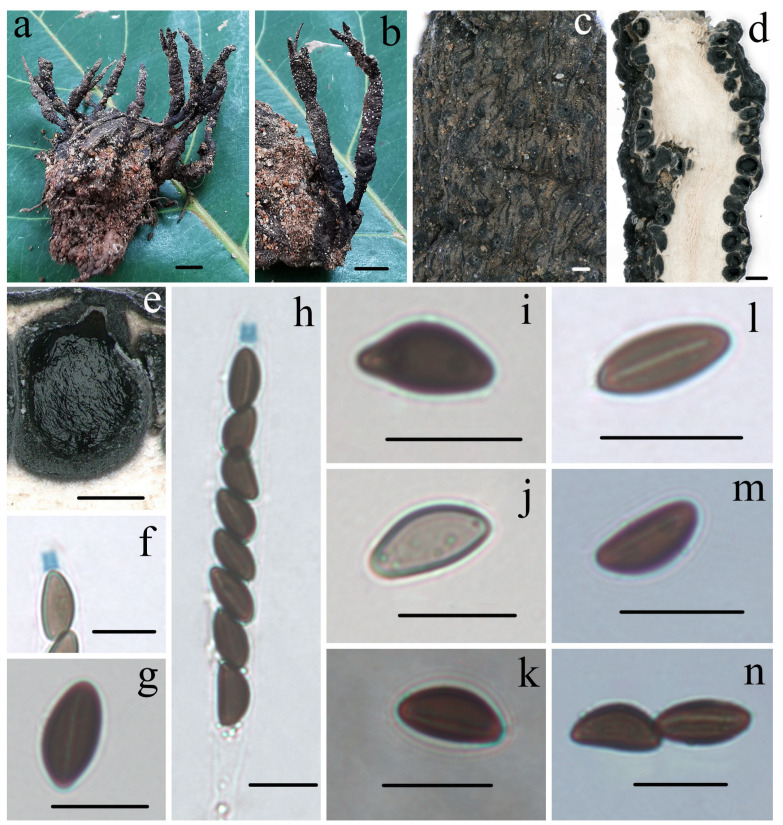
*Heteroxylaria terminaliicola* (FCATAS 921, holotype). (**a**,**b**) Stromata on fallen fruit. (**c**) Stromatal surface. (**d**,**e**) Section through stroma, showing perithecia. (**f**) Ascal apical ring in Melzer’s reagent. (**g**) Ascospore with germ slit in KOH. (**h**) Asci with ascal apical ring in Melzer’s reagent. (**i**) Ascospore with beaked ends in 5% KOH. (**j**) Ascospore in 5% KOH. (**k**) Ascospore in India ink. (**l**) Ascospore with germ slit in Melzer’s reagent. (**m**) Ascospore in 1% SDS. (**n**) Ascospores with germ slit in 1% SDS. Scale bars: (**a**,**b**) = 1 cm; (**c**,**e**) = 200 µm; (**d**) = 500 µm; and (**f**–**n**) = 10 µm.

***Heteroxylaria terminaliicola*** Hai X. Ma, A.H. Zhu and Y. Li, sp. nov. Figure 5 and Figure 7j,k.

**MycoBank:** MB854837

**Etymology:** *terminaliicola* (Lat.): referring to the host genus *Terminalia* which the fungus inhabits.

**Holotype:** CHINA: Hainan Province, Haikou City, Chinese Academy of Tropical Agricultural Sciences, on nutshells of *Terminalia catappa* L. (Combretaceae), 20 November 2020, Ma Haixia, FCATAS921 (Col.26), GenBank numbers: ITS: MZ648854, RPBS: MZ707125, TUB: MZ695802, LSU: MZ703410.

**Description:** *Sexual morph*: *Stromata* upright, solitary or sometimes clustered, straight to curved, unbranched or branched at apices or fertile parts, apically attenuated into a mucronate or blunt sterile apex, 2.5–7 cm total height; fertile parts 10–20 mm high × 1.5–3.5 mm broad, cylindrical, surface dark brown to blackish, with inconspicuous perithecial mounds, occasionally with dark brown tomentum in part, eventually black outer layer splitting longitudinally into stripes; the stipes 13–50 mm high × 1.0–6.0 mm broad, terete to rarely flattened, often contorted, ill-defined, glabrous, the pannose base swollen and slightly tomentose; surface black, roughened with perithecia and tomentose; interior white, solid, and woody. *Perithecia* subglobose, 350–600 µm. Ostioles papillate. *Asci* eight-spored arranged in uniseriate manner, cylindrical, long-stipitate, (95–)105–155(–170) µm total length, the spore-bearing parts (55–)65–75(–100) µm long × (6.4–)7.0–8.0(–9.0) µm broad, the stipes 35–70 µm long, with apical ring bluing in Melzer’s reagent, tubular to short tubular, 2.2–3.8 µm high × 2.4–3.2 µm diam. *Ascospores* brown, unicellular, fusiform or navicular, inequilateral, with broadly rounded ends, one end slightly pinched sometimes, or beaked occasionally, smooth, (10.0–)11–13.0(–13.8) × (5.2–)6.0–7.0(–7.8) µm (M = 12.0 × 6.7 µm, Q = 1.8, n = 60/2), with a conspicuous straight germ slit slightly less than spore-length, lacking a sheath or appendages visible in India ink or 1% SDS.

**Additional specimens examined:** CHINA: Hainan Province, Haikou City, Chinese Academy of Tropical Agricultural Sciences, on nutshells of T. catappa, 20 November 2020, Ma HaiXia, FCATAS922 (Col.27), GenBank numbers: ITS: MZ648855, RPBS: MZ707126, TUB: MZ695803, LSU: MZ703411.

**Notes:** *Heteroxylaria terminaliicola* is distinguished by its strong stromata with inconspicuous perithecial mounds, large tubular apical ring, brown ascospores with a conspicuous straight germ slit, and grows on nutshells of *T. catappa*. Pande and Waingankar (2004) [77] described two *Xylaria* species on fallen fruits of *Terminalia* from Western India. *Xylaria terminaliae-bellericae* Pande and Waingankar was found on fallen fruits of *T. bellerica*, and *X. terminaliae-crenulatae* Pande and Waingankar on fallen fruits of *T. crenulata* [77]. However, *H. terminaliicola* is distinctly different from the two species. *Xylaria terminaliae*-*bellericae* has hairy stipe, more acute sterile apex, and slightly smaller ascospores 8–11 × 3–5.4 µm, whereas *X. terminaliae-crenulatae* has thinner, filiform, unbranched stromata, and larger ascospores 10.5–15.8 × 5.3–10.5 µm [77].

*Heteroxylaria terminaliicola* somewhat resembles *H. oxyacanthae* by sharing stromatal morphology, but differs from the latter in having stromata with apices often shriveled or broken, salmon buff to dark brown, slightly smaller ascospores (9.5–)10–11.5(–12) × (4–)4.5–5.5(–6) µm (M = 10.8 × 5.0 µm), and grows on seeds of *C. oxyacantha* (Rosaceae) [56,70,73]. *Xylaria rhizocola* (Mont.) Fr. is also similar to *H. terminaliicola* in ascospores dimensions, but differs in having rounded stromatal tip, surface non-tomentum, inverted hat-shaped and slightly smaller apical ring 1.5–2 µm high × 2 µm broad, and grows on the buried seed of an unknown host [56]. The phylogenetic trees show that *H. terminaliicola* and *H. reevesiae* Y.M. Ju, J.D. Rogers and H.M. Hsieh are closely related, but the latter differs in having conspicuous perithecial mounds, a smaller and inverted hat-shaped apical ring 1.5–2 µm high × 2–2.5 µm broad, smaller ascospores (8.5–)9–10.5(–11) × (4–)4.5–5.5(–6) µm (M = 9.7 × 5.0 µm), and grows on fruits of *R. formosana* (Sterculiaceae) [56].

**Figure 6:**
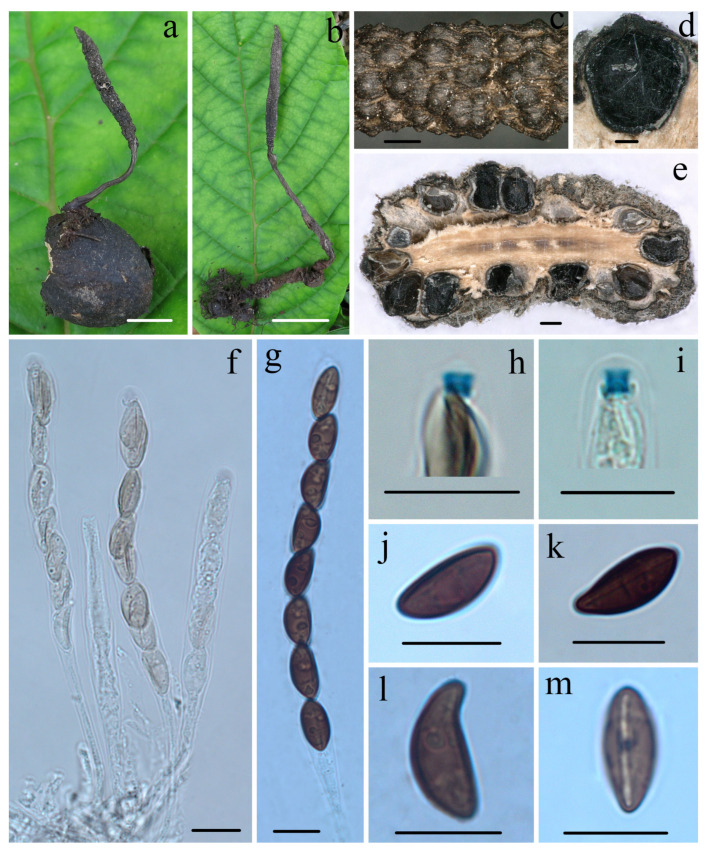
*Heteroxylaria oxyacanthae* (FCATAS 906). (**a**,**b**) Stroma on fallen fruit. (**c**) Stromatal surface. (**d**,**e**) Section through stroma, showing perithecia. (**f**) Young asci in water. (**g**) Asci in India ink. (**h**,**i**) Ascal apical ring in Melzer’s reagent. (**j**) Ascospore in water. (**k**) Ascospore in Melzer’s reagent. (**l**) Ascospore with beaked ends in India ink. (**m**) Ascospore with germ slit in 1% SDS. Scale bars: (**a**,**b**) = 1 cm; (**c**) = 500 µm; (**d**,**e**) = 100 µm; and (**f**–**m**) = 10 µm.

**Figure 7:**
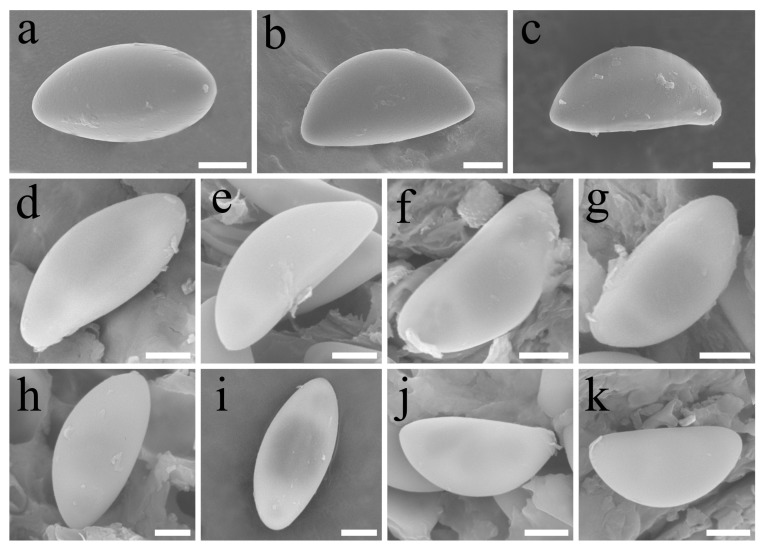
(**a**–**c**) *Heteroxylaria juglandicola* (FCATAS 3667, holotype); (**d**,**e**) *Heteroxylaria cordiicola* (FCATAS 907, holotype); (**f**,**g**) *Heteroxylaria meliicola* (FCATAS 869, holotype); (**h**,**i**) *Heteroxylaria oxyacanthae* (FCATAS 906); and (**j**,**k**) *Heteroxylaria terminaliicola* (FCATAS 921, holotype). Scale bars: 2 µm.

***Heteroxylaria oxyacanthae*** (Tul. and C. Tul.) Hai X. Ma, A.H. Zhu and Y. Li, comb. nov. Figure 6 and Figure 7h,i

**MycoBank:** MB854838

**Basionym:** *Xylaria oxyacanthae* Tul. and C. Tul., Selecta Carpologia Fungorum 2, p.15.1863.

**Holotype:** FRANCE: Bethisy, on seeds of *Crataegus oxyacantha* (Rosaceae), Jun 1860, Tulasne, R. (holotype PC 0096746!).

**Description:** *Sexual morph*: Stromata upright or prostrate, unbranched or branched from the fertile parts, 3–13 cm total height, long-stipitate; fertile parts 6–40 mm high × 1.5–8.0 mm broad, fusiform to cylindrical or forked, sometimes flattened, with acute grey-white sterile apices up to 2 mm long, exterior smooth at young stromata, white to cream-coloured, mature stromata black, strongly nodulose, with gray-white peeling outer layer, interior yellow to light brown, solid, and woody; stipes 27–90 mm high × 0.5–3.5 mm broad, smooth to downy, terete, sometimes flattened, usually contorted, with longitudinally wrinkles, arising from a pannose, slightly enlarged base. Surface roughened with wrinkles and perithecial contours. Perithecia subglobose, 300–400 µm. Ostioles papillate. Asci eight-spored arranged in uniseriate manner, cylindrical, long-stipitate, 110–185 µm total length, the spore-bearing part (60–)65–75(–80) µm long × 6–7 µm broad, with apical ring bluing in Melzer’s iodine reagent, more or less rectangular or discoid, 1.8–2.5 µm high × 2–2.5 µm broad. Ascospores brown to dark brown, unicellular, ellipsoid-inequilateral, with narrowly to broadly rounded ends, smooth, (10–)11–12(–12.5) × (4.5–)5.0–5.5(–6) µm (M = 11.0 × 5.1 µm, Q = 2.2, n = 60/2), with straight germ slit full-length or nearly so, lacking a sheath or appendages in India ink or 1% SDS.

**Specimens examined:** CHINA: Jilin Province, Changchun City, Jingyuetan Forest Park, on seeds of Crataegus maximowiczii Schneid (Rosaceae), 1 September 2014, Ma Haixia FCATAS905 (Col.130), GenBank numbers: ITS: MZ620654, RPBS: MZ678635, TUB: MZ695789, LSU: MZ703199; FCATAS906 (Col.132), GenBank numbers: ITS: MZ620655, RPBS: MZ678636, TUB: MZ695790, LSU: MZ703200.

**Notes:** *Xylaria oxyacanthae* was originally described on seeds of *C. oxyacantha* L. (Rosaceae) from France. The species is characterized by cylindrical to irregular stromata with short acute sterile apices on tomentose stipes, surface blackish with a gray to brown peeling outer layer, and brown to dark brown ascospores with straight germ slit [56,70,73]. We collected the two present materials which both were found on nuts of *C. maximowiczii* from Jilin province of northeastern China. We follow Ju et al. (2018) [56] who determined the type specimen of *X. oxyacanthae* on seeds of *C. oxyacantha* (Rosaceae) from France. Based on morphological studies and phylogenetic analyses, we determined that the Chinese collections are conspecific with *X. oxyacanthae* and transfer it to *Heteroxylaria*.

***Heteroxylaria palmicola*** (G. Winter) Hai X. Ma, A.H. Zhu and Y. Li, comb. nov.

**MycoBank:** MB854839

**Basionym:** *Xylaria palmicola* G. Winter, Grevillea 15:89. 1887.

**Holotype:** BRAZIL: Pr. St. Catharina, São Francisco, on seeds of Euterpe sp. (Arecaceae), May 1885, Ule, E. 353 (holotype HBG!)

For a detailed description of *Xylaria palmicola*, see Dennis (1956) [74].

**Notes:** *Xylaria palmicola* was introduced by G. Winter (1887) from seeds of *Euterpe* sp. (Arecaceae) in Brazil. This species is characterized by slender, cylindrical, and long stroma, simple, with pointed apex, perithecia rather prominent, brown surface with longitudinally along numerous and closely spaced parallel cracks, stipes defined well, very long, becoming longitudinally wrinkled and some twisted when dried, and ascospores with pointed ends [74]. There is no sequence data of the type of *X. palmicola*, but a putatively named collection (PDD604) from New Zealand [27]. In the phylogenetic tree, the strain from New Zealand distributed in the genus *Heteroxylaria* (Figure 1). Based on morphological studies and phylogenetic analyses, we propose the transfer of *X. palmicola* into *Heteroxylaria*.

***Heteroxylaria reevesiae*** (Y.M. Ju, J.D. Rogers and H.M. Hsieh) Hai X. Ma, A.H. Zhu and Y. Li, comb. nov.

**MycoBank:** MB854840

**Basionym:** *Xylaria reevesiae* Y.M. Ju, J.D. Rogers and H.M. Hsieh, Mycologia 110(4): 742. 2018.

**Holotype:** CHINA: Pingtung Co., Hengchun, Kenting, on fallen fruits of Reevesia formosana (Sterculiaceae), 16 July 2001, Ju YM and Hsieh HM, 90,071,609 (holotype HAST).

For a detailed description of *Xylaria reevesiae*, see Ju et al. (2018) [56].

**Notes:** *Xylaria reevesiae* was introduced by Y.M. Ju, J.D. Rogers, and H.M. Hsieh (2018) [56] from fallen fruits of *Reevesia formosana* (Sterculiaceae) in Taiwan of China. This species is characterized by cylindrical stroma with apiculate to acicular apices on glabrous or tomentose stipe, dark brown to blackish surface with conspicuous perithecial mounds, an overlain narrowly striped outermost layer, ascospores brown, smooth, with a straight germ slit. In the phylogenetic tree, the strain from the type specimen distributed in the genus *Heteroxylaria* (Figure 1). Based on morphological studies and phylogenetic analyses, we propose the transfer of *X. reevesiae* into *Heteroxylaria*.

***Heteroxylaria rohrensis*** (Friebes, A. Gallé, H.M. Hsieh and Y.M. Ju) Hai X. Ma, A.H. Zhu and Y. Li, comb. nov.

**MycoBank:** MB854841

**Basionym:** *Xylaria rohrensis* Friebes, A. Gallé, H.M. Hsieh and Y.M. Ju, Nova Hedwigia 115(1-2): 137. 2022.

**Holotype:** AUSTRIA: Styria, Rohr an der Raab, Südoststeiermark, on nutshells of Juglans regia, 3 August 2020, Gallé, A. (holotype HAST 145766).

For a detailed description of *Xylaria rohrensis*, see Friebes et al. (2022) [57].

**Notes:** *Xylaria rohrensis* was introduced by Friebes, A. Gallé, H.M. Hsieh and Y.M. Ju (2022) [57] from nutshells of *Juglans regia* (Juglandaceae) in Austria. The species is characterized by cylindrical stromata unbranched or branched once or twice at clavae, asci with an apical ring inverted hat-shaped, and brown to dark brown ascospores ellipsoid-inequilateral, with a straight germ slit [57]. Based on morphological studies and phylogenetic analyses, we propose the transfer of *X. rohrensis* into *Heteroxylaria*.

Notes on doubtful species:

***Xylaria putaminum*** Maire and Durieu

This fungus is a invalid species for lacking the Latin description and the type specimens [57,75]. Based on morphological studies and sequence analyses in the study, it may be conspecific with *H. palmicola*, further identification should be made. Therefore, the species was as an uncertain species in *Heteroxylaria*.

## 4. Discussion

In the present study, the genus *Heteroxylaria* represented by *H. oxyacanthae*, *H. cordiicola*, *H. juglandicola*, *H. meliicola*, *H. palmicola*, *H. reevesiae*, *H. rohrensis*, and an uncertained species (*X. putaminum*), is separated from *Xylaria* within Xylariaceae from a phylogenetic perspective and ecological characters in the present study. The new genus *Heteroxylaria* is mainly characterized by cylindrical stromata, brown ascospores, and grows on the nutshell of fruits, closely related to *Hypocreodendron*. However, the genus *Hypocreodendron* has obvious differences in stromatal and ascospore morphology, and grows on ant nests [71].

*Xylaira* is the largest genus in Xylariaceae including 879 taxa recorded in Index Fungorum (http://www.indexfungorum.org/, accessed on 30 January 2024). The genus *Xylaria* in the current taxonomic concept clearly indicated to be not monophyletic [24,27,57,78], which was once more confirmed in this paper. The phylogenies generated in different studies suggested that a polyphasic taxonomic approach for reconstructing classification system of *Xylaria* was needed. Therefore, we carried out morphological, ecological, and sequence data to erect a polyphasic characterization of a separate clade (HD clade) phylogenetically resolved inside *Xylaria*, including taxa closely related to *X. oxyacanthae*, for which we present the new genus *Heteroxylaria*, sharing many morphological features with *Xylaria* in the traditional discriminative criteria. This is not unique for the genus *Xylaria*, but also similar for some other genera of Ascomycota and Basidiomycota; for example, several genera were separated from *Hypoxylon* within Hypoxylaceae [20,79] and the phylogenetic updates in Agaricales with an emphasis on *Tricholomopsis* [80]. Currently, the placement of other clades (TE, HY, PO, and IA) in *Xylaria* remain unresolved, and therefore, the polyphasic taxonomic approaches based on morphological, chemotaxonomic, and phylogenetic data including more gene sequences or genome sequencing for resolving the confusion of *Xylaria* species associated with other fruits or substances are needed in the further studies.

*Xylaria* are an extremely diverse genus of fungi, many taxa have the ability to decompose cellulose, hemicellulose, lignin, and carbohydrates in the substrate [81,82,83]. Some studies suggested that *Xylaria* species may have a preference for certain types of lignin over others for some degradative enzymes [84,85,86,87,88]. *Heteroxylaria* taxa grow on the nutshells of fruits or seeds, and whether the degrading enzymes can be the key evidence for resolving the taxonomic confusion of *Xylaira* remains to be explored in the future.

## Figures and Tables

**Table 1:** List of taxa used for the phylogenetic reconstruction. GenBank accession numbers, specimen numbers, origin, and reference studies are given. Holotype specimens are labelled with HT. Species highlighted in bold were derived from this study. N/A: not available.

Speices	Specimen No.	Origin	Host	GenBank Accession Number			References
				ITS	RPB2	ß-Tubulin	
*Amphirosellinia fushanensis*	HAST91111209(HT)	China	dead twigs	GU339496	GQ848339	GQ495950	[27,49]
*A. nigrospora*	HAST91092308(HT)	China	dead twigs	GU322457	GQ848340	GQ49595	[27,49]
*Astrocystis bambusae*	HAST89021904	China	bamboo culms	GU322449	GQ844836	GQ495942	[27]
*As. mirabilis*	HAST94070803	China	bamboo culms	GU322448	GQ844835	GQ49594	[27]
*Barrmaelia rappazii*	CBS142771(HT)	Norway	twigs and branches of *Populus tremula*	MF488989	MF488998	MF489017	[50]
*Brunneiperidium gracilentum*	MFLUCC14-0011(ET)	Italy	twigs of *Tamarix gallica*	KP297400	KP340528	KP406611	[51]
*B. involucratum*	MFLUCC14-0009(ET)	Italy	cone of *Pinus sylvestris*	KP297399	KP340527	KP406610	[51]
*Clypeosphaeria mamillana*	CBS140735(ET)	France	branches of *Cornus alba*	KT949897	MF489001	MH704637	[50,52]
*Collodiscula bambusae*	GZUH0102(HT)	China	stalk of bamboo	KP054279	KP276675	KP276674	[53]
*C. fangjingshanensis*	GZUH0109(HT)	China	culm of bamboo	KR002590	KR002592	KR002589	[54]
*Daldinia loculatoides*	CBS113279(PT)	UK	*Fagus* sp.	MH862918	KY624247	KX271246	[20,55]
*Entoleuca mammata*	JDR100	France	*Fagus* sp.	GU300072	GQ844782	GQ470230	[27]
*Entosordaria perfidiosa*	CBS142773(HT)	Austria	bark of old trunks of living *Acer pseudoplatanus*	MF488993	MF489003	MF489021	[50]
*Euepixylon sphaeriostoma*	JDR261	USA	*Fraxinus* wood	GU292821	GQ844774	GQ470224	[27]
***Heteroxylaria cordiicola***	**FCATAS907(HT)**	**China**	**nutshells of *Cordia dichotoma***	**MZ648852**	**MZ707116**	**MZ695791**	**This study**
***H. cordiicola***	**FCATAS908**	**China**	**nutshells of *Cordia dichotoma***	**MZ648853**	**MZ707117**	**MZ695792**	**This study**
***H. juglandicola***	**FCATAS3667(HT)**	**China**	**nutshells of *Juglans regia***	**PQ009296**	**PQ010279**	**PQ010278**	**This study**
***H. juglandicola***	**FCATAS3668**	**China**	**nutshells of *Juglans regia***	**PQ009297**	**N/A**	**N/A**	**This study**
***H. meliicola***	**FCATAS869(HT)**	**China**	**nutshells of *Melia toosendan***	**MZ648845**	**MZ707104**	**MZ695773**	**This study**
***H. meliicola***	**FCATAS870**	**China**	**nutshells of *Melia toosendan***	**MZ648846**	**N/A**	**N/A**	**This study**
***H. oxyacanthae***	**FCATAS905**	**China**	**nuts of *Crataegus maximowiczii***	**MZ620654**	**MZ678635**	**MZ695789**	**This study**
***H. oxyacanthae***	**FCATAS906**	**China**	**nuts of *Crataegus maximowiczii***	**MZ620655**	**MZ678636**	**MZ695790**	**This study**
*H. oxyacanthae*	JDR859	USA	seeds of *Crataegus monogyna*	GU322434	GQ844820	GQ495927	[27]
*H. oxyacanthae*	YMJ1184	Germany	seeds of *Carpinus betulus*	MF773430	MF773434	MF773438	[27,56]
*H. oxyacanthae*	YMJ1320	Germany	Fruits of *Cornus sanguinea*	MF773431	MF773435	MF773439	[27,56]
*H. oxyacanthae*	YMJ1660	France	plum seeds of *Prunus* sp.	MF773429	MF773433	MF773437	[27,56]
*H. palmicola*	PDD604	New Zealand	fruits of palm	GU322436	GQ844822	GQ495929	[27]
*H. reevesiae*	HAST90071609(HT)	China	fruits of *Reevesia formosana*	GU322435	GQ844821	GQ495928	[27]
*H. rohrensis*	HAST145766(HT)	Austria	nutshells of *Juglans regia*	ON261187	ON262774	ON262768	[57]
*H. rohrensis*	HAST145767	Austria	nutshells of *Juglans regia*	ON261188	ON262775	ON262769	[57]
*Heteroxylaria* sp.	HAST145770	Spain	buried stones of *Olea europea* var. *sylvestris*	ON248209	ON262776	ON262770	[57]
***H. terminaliicola***	**FCATAS921(HT)**	**China**	**nutshells of *Terminalia catappa***	**MZ648854**	**MZ707125**	**MZ695802**	**This study**
***H. terminaliicola***	**FCATAS922**	**China**	**nutshells of *Terminalia catappa***	**MZ648855**	**MZ707126**	**MZ695803**	**This study**
*Hypocreodendron sanguineum* (*Dixcoxylaria myrmecophila*)	JDR169	Mexico	nests of *Atta mexicana*	GU322433	GQ844819	GQ487710	[27]
*Hypoxylon fragiforme*	MUCL51264(ET)	Germany	dead stump of *Fagus sylvatica*	KC477229	KM186296	KX271282	[20,51,58]
*Kretzschmaria deusta*	CBS163.93	Germany	wood	KC477237	KY624227	KX271251	[58]
*K. guyanensis*	HAST89062903	China	bark	GU300079	GQ844792	GQ478214	[27]
*K. neocaledonica*	HAST94031003	China	bark	GU300078	GQ844788	GQ478213	[27]
*Nemania abortiva*	BiSH467(HT)	USA	decayed angiosperm wood	GU292816	GQ844768	GQ470219	[27]
*N. diffusa*	HAST91020401	China	bark	GU292817	GQ844769	GQ470220	[27]
*Neoxylaria arengae*	MFLUCC15-0292	Thailand	dead petiole of *Arenga pinnata*	MT496747	MT502418	N/A	[24]
*Podosordaria mexicana*	WSP176	Mexico	horse dung	GU324762	GQ853039	GQ844840	[27]
*P. muli*	WSP167(HT)	Mexico	mule dung	GU324761	GQ853038	GQ844839	[27]
*Poronia pileiformis*	WSP88113001(ET)	China	cow dung	GU324760	GQ853037	GQ502720	[27]
*Rosellinia buxi*	JDR99	France	*Buxus sempervivens*	GU300070	GQ844780	GQ470228	[27]
*R. merrillii*	HAST89112601	China	bark	GU300071	GQ844781	GQ470229	[27]
*R. sanctacruciana*	HAST90072903	China	fronds of *Arenga engleri*	GU292824	GQ844777	GQ470227	[27]
*Sarcoxylon compunctum*	CBS359.61	South Africa	N/A	KT281903	KY624230	KX271255	[28]
*Stilbohypoxylon elaeidis*	MFLUCC15-0295a	Thailand	dead petiole of *Elaeis guineensis*	MT496745	MT502416	MT502420	[24]
*S. elaeidicola*	YMJ173	French Guiana	palm	EF026148	GQ844826	EF025616	[27,59]
*S. quisquiliarum*	YMJ172	French Guiana	wood	EF026119	GQ853020	EF025605	[27,59]
*Xylaria adscendens*	HAST570	Guadeloupe	wood	GU300101	GQ844817	GQ487708	[27]
*X. aethiopica*	YMJ1136	Ethiopia	pods of *Millettia ferruginea*	MH790445	MH785222	MH785221	[60]
*X. allantoidea*	HAST94042903	China	trunk	GU324743	GQ848356	GQ502692	[27]
*X. amphithele*	HAST529	Guadeloupe	dead leaves	GU300083	GQ844796	GQ478218	[27]
*X. apoda*	HAST90080804	China	bark	GU322437	GQ844823	GQ495930	[27]
*X. arbuscula*	HAST89041211	China	bark	GU300090	GQ844805	GQ478226	[27]
*X. atrosphaerica*	HAST91111214	China	bark	GU322459	GQ848342	GQ495953	[27]
*X. berteri*	HAST90112623	China	wood	GU324749	GQ848362	AY951763	[27]
*X. brunneovinosa*	HAST720(HT)	China	ground of bamboo plantation	EU179862	GQ853023	GQ502706	[27,61]
*X. cirrata*	HAST664(ET)	China	ground of vegetable farm	EU179863	GQ853024	GQ502707	[27,61]
*X. cranioides*	HAST226	China	wood	GU300075	GQ844785	GQ478210	[27]
*X. cubensis*	JDR860	USA	wood	GU991523	GQ848365	GQ502700	[27]
*X. culleniae*	JDR189	Thailand	pod	GU322442	GQ844829	GQ495935	[27]
*X. curta*	HAST92092022	China	bark	GU322443	GQ844830	GQ495936	[27]
*X. digitata*	HAST919	Ukraine	wood	GU322456	GQ848338	GQ495949	[27]
*X. enterogena*	HAST785	French Guiana	wood	GU324736	GQ848349	GQ502685	[27]
*X. escharoidea*	HAST658(ET)	China	ground of mango orchard	EU179864	GQ853026	GQ502709	[27]
*X. fabacearum*	MFLU16-1061(HT)	Thailand	seed pods of Fabaceae	NR171104	MT212202	MT212220	[62]
*X. fabaceicola*	MFLU16-1072(HT)	Thailand	seed pods of Fabaceae	NR171103	MT212201	MT212219	[62]
*X. feejeensis*	HAST92092013	China	bark	GU322454	GQ848336	GQ495947	[27]
*X. fimbriata*	HAST491	Martinique	termite nest	GU324753	GQ853022	GQ502705	[27]
*X. fissilis*	HAST367	Martinique	bark	GU300073	GQ844783	GQ470231	[27]
*X. frustulosa*	HAST92092010	China	bark	GU322451	GQ844838	GQ495944	[27]
*X.* cf. *glebulosa*	HAST431	Martinique	Fruits of *Swietenia macrophylla*	GU322462	GQ848345	GQ495956	[27]
*X. globosa*	HAST775	Guadeloupe	bark	GU324735	GQ848348	GQ502684	[27]
*X. grammica*	HAST479	China	wood	GU300097	GQ844813	GQ487704	[27]
*X. griseosepiacea*	HAST641(HT)	China	ground of mango orchard	EU179865	GQ853031	GQ502714	[27,61]
*X. haemorrhoidalis*	HAST89041207	China	bark	GU322464	GQ848347	GQ502683	[27]
*X. hypoxylon*	HAST95082001	China	wood	GU300095	GQ844811	GQ487703	[27]
*X. ianthinovelutina*	HAST553	Martinique	fruit of *Swietenia macrophylla*	GU322441	GQ844828	GQ495934	[27]
*X. intracolorata*	HAST90080402	China	bark	GU324741	GQ848354	GQ502690	[27]
*X. intraflava*	HAST725(HT)	China	ground of bamboo plantation	EU179866	GQ853035	GQ502718	[27]
*X. juruensis*	HAST92042501	China	*Arenga engleri*	GU322439	GQ844825	GQ495932	[27]
*X. karyophthora*	DRH059	Guyana	seeds of *Chlorocardium* sp.	KY564220	KY564216	N/A	[63]
*X. laevis*	HAST95072910	China	bark	GU324747	GQ848360	GQ502696	[27]
*X. lindericola*	FCATAS852	China	leaves of *Lindera robusta*	MZ005635	MZ031982	MZ031978	[43]
*X. liquidambaris*	HAST93090701	China	fruits of *Liquidambar formosana*	GU300094	GQ844810	GQ487702	[27]
*X. liquidambaris*	FCATAS874	China	fruits of *Liquidambar formosana*	MZ620275	MZ707107	MZ695775	[64]
*X. meliacearum*	JDR148	Puerto Rico	petioles and infructescence of *Guarea guidonia*	GU300084	GQ844797	GQ478219	[27]
*X. microceras*	HAST414	Guadeloupe	wood	GU300086	GQ844799	GQ478221	[27]
*X. montagnei*	HAST495	Martinique	wood	GU322455	GQ848337	GQ495948	[27]
*X. multiplex*	JDR259	USA	wood	GU300099	GQ844815	GQ487706	[27]
*X. muscula*	HAST520	Guadeloupe	dead branch	GU300087	GQ844800	GQ478222	[27]
*X. nigripes*	HAST653	China	ground of mango orchard	GU324755	GQ853027	GQ502710	[27]
*X. ochraceostroma*	HAST401(HT)	China	ground of mango orchard	EU179869	GQ853034	GQ502717	[27,61]
*X. oligotoma*	HAST784	French Guiana	wood	GU300092	GQ844808	GQ487700	[27]
*X. ophiopoda*	HAST93082805	China	bark	GU322461	GQ848344	GQ495955	[27]
*X. papulis*	HAST89021903	China	wood	GU300100	GQ844816	GQ487707	[27]
*X. phyllocharis*	HAST528	Guadeloupe	dead leaves	GU322445	GQ844832	GQ495938	[27]
*X. plebeja*	HAST91122401	China	trunk of *Machilus zuihoensis*	GU324740	GQ848353	GQ502689	[27]
*X. polymorpha*	JDR1012	USA	wood	GU322460	GQ848343	GQ495954	[27]
*X. polysporicola*	FCATAS848(HT)	China	leaves of *Polyspora hainanensis*	MZ005592	MZ031980	MZ031976	[43]
*X. regalis*	HAST920	India	log of *Ficus racemosa*	GU324745	GQ848358	GQ502694	[27]
*X. rogersii*	FCATAS915(HT)	China	fruits of *Magnolia* sp.	MZ648827	MZ707121	MZ695800	[31]
*X. schimicola*	FCATAS896(HT)	China	fruits of *Schima noronhae*	MZ648850	MZ707114	MZ695787	[31]
*X. schweinitzii*	HAST92092023	China	bark	GU322463	GQ848346	GQ495957	[27]
*X. scruposa*	HAST497	Martinique	wood	GU322458	GQ848341	GQ495952	[27]
*X. sicula*	HAST90071613	China	fallen leaves	GU300081	GQ844794	GQ478216	[27]
*Xylaria* sp. 6	JDR258	USA	leaves of *Tibouchina semidecandra*	GU300082	GQ844795	GQ478217	[27]
*X. striata*	HAST304	China	branch of *Punica granatum*	GU300089	GQ844803	GQ478224	[27]
*X. telfairii*	HAST90081901	China	bark	GU324738	GQ848351	GQ502687	[27]
*X. theaceicola*	FCATAS903(HT)	China	fruits of *Schima villosa*	MZ648848	MZ707115	MZ695788	[31]
*X. tuberoides*	HAST475	Martinique	wood	GU300074	GQ844784	GQ478209	[27]
*X. venustula*	HAST 88113002	China	bark	GU300091	GQ844807	GQ487699	[27]
*X. vivantii*	HAST519(HT)	Martinique	fruits of *Magnolia* sp.	GU322438	GQ844824	GQ495931	[27]
*X. wallichii*	FCATAS923(HT)	China	fruits of *Schima wallichii*	MZ648861	MZ707118	MZ695793	[31]

## Data Availability

Publicly available datasets were analyzed in this study. All newly generated sequences were deposited in GenBank (https://www.ncbi.nlm.nih.gov/genbank/; accessed on 13 July 2024; Table 1). All new taxa were deposited in MycoBank (https:www.mycobank.org/; accessed on 18 July 2024; MycoBank identifiers follow new taxa and new combinations).
